# Oil Road Effects on the Anuran Community of a High Canopy Tank Bromeliad (*Aechmea zebrina*) in the Upper Amazon Basin, Ecuador

**DOI:** 10.1371/journal.pone.0085470

**Published:** 2014-01-08

**Authors:** Shawn F. McCracken, Michael R. J. Forstner

**Affiliations:** 1 Department of Biology, Texas State University, San Marcos, Texas, United States of America; 2 The TADPOLE Organization, San Marcos, Texas, United States of America; University of Kent, United Kingdom

## Abstract

Tropical forest canopies are among the most species-rich terrestrial habitats on earth and one of the remaining relatively unexplored biotic frontiers. Epiphytic bromeliads provide microhabitat for a high diversity of organisms in tropical forest canopies and are considered a keystone resource. A number of amphibians inhabit these phytotelmata, yet their ecological role and status in forest canopies remains unknown. For this study, anurans were collected from an upper canopy tank bromeliad (*Aechmea zebrina*) at ∼20–45 m (x¯ = 33 m) above the forest floor. Bromeliads were sampled from trees located near trails in undisturbed primary rainforest and oil access roads in the Yasuní Biosphere Reserve of Amazonian Ecuador. We collected 95 anurans representing 10 species from 160 bromeliads in 32 trees. We used generalized linear mixed models to assess the effects of disturbance and habitat factors on the occupancy and abundance of anurans collected. Bromeliads in forest along oil roads had a lower occupancy and abundance of anurans than those in undisturbed forest, a somewhat unexpected result due to the intactness and quality of forest adjacent to the roads. Recorded habitat variables had no relationship with occupancy or abundance of anurans, and did not differ significantly between treatments. Our findings reveal that even the minimal footprint of natural resource extraction operations, primarily roads, in rainforest environments can have significant negative impacts on the unique upper canopy anuran community. Based on these results, we recommend that natural resource development treat rainforest habitat as an offshore system where roads are not used, employ industry best practice guidelines, and current access roads be protected from colonization and further deforestation.

## Introduction

The upper canopy of tropical forests are a relatively unexplored biotic frontier, yet are among the most species-rich terrestrial habitats on Earth, supporting up to 50% of described extant species [1], [2]. A diversity of microhabitats available in the canopy creates unique ecological niches structurally supporting high species richness of arboreal communities [3], [4]. A key component of tropical rainforest canopies are phytotelmata, defined as plants or parts of plants that hold rainwater (e.g. bromeliads, inflorescences, and tree holes) [5]. In some moist tropical locations bromeliads impound up to 50,000 liters of water per hectare [6], creating a 3-dimensional “wetland in the sky” [7]. In particular, epiphytic tank bromeliads hold a large volume of water and are a keystone resource for invertebrates, vertebrates, and other plants [8], [9]. Bromeliads have been reported to have incredibly high biodiversity in previous arthropod surveys [6], yet most research was completed without ever actually entering the canopy [10]. Indeed, research in the canopy is still a relatively new discipline facilitated by recent advances in canopy access techniques [11]. Thus far, canopy research has largely focused on arthropods, birds, mammals, plants, and ecological processes [12]; investigations of upper canopy herpetofauna have only recently been reported [13]–[15].

Habitat loss is the single greatest threat to worldwide amphibian diversity [16]. Ecuador has the highest deforestation rate (28.6% of 1990 forest area lost by 2010) and one of the worst environmental records in South America [17], [18]. In the Ecuadorian Amazon, petroleum operations have been the driving force of deforestation and pollution [19]. Physical alterations of environments, such as road building for access to oilfields, directly contribute to deforestation [20]. Roads and other linear clearings (e.g., pipelines and power lines) are rapidly expanding in tropical rainforests with known negative impacts to the habitat and ecosystem [21]. Beyond deforestation, the negative impacts of linear clearings (particularly roads) in tropical rainforests include edge effects, faunal intrusions, physical disturbance, road-related mortality, barrier effects, and pollution [21], [22]. These roads allow access and enable settlers to exploit these regions with agriculture, hunting, logging, and mining operations causing even greater environmental degradation [20], [23]. Agricultural colonization by small-scale farmers following oil roads and pipeline paths has resulted in a ∼2% per year deforestation rate in the Ecuadorian Amazon, greater than any other Amazon nation [24], [25]. Finer et al. [19] reported that a minimum of 180 oil and gas blocks covered ∼688 000 km^2^ of forest in the western Amazon, and this number is increasing. The development of these blocks will require forest clearing for exploratory and extraction activities. The rapid exploitation of natural resources in the Ecuadorian Amazon has already had a profound effect on the forest and its indigenous inhabitants [20]. Yet, little is known about consequences of these anthropogenic changes on canopy biota.

Canopy properties, both biotic and abiotic, are influenced by anthropogenic disturbance [2], [26]. Determining which factors are affected by disturbance is a fundamental goal of conservation ecology [27]. In tropical ecosystems, identifying effects of these factors are complicated by a large number of undescribed species in co-occurring species assemblages [5], [28]. Community ecologists often avoid these complications by restricting their studies to a single taxonomic group at the family or guild level [29]–[33], thus potentially biasing our understanding of patterns and processes in complete ecological communities [34], [35]. Tank bromeliads provide a model system for evaluating complete communities with a taxonomically rich fauna living in a structurally discrete habitat [5]. Sampling the complete anuran community of an epiphytic canopy tank bromeliad in conjunction with measures of habitat variables across differing forest disturbance levels provides an opportunity to identify both natural and anthropogenic factors influencing species assemblages in an ecologically defined natural community.

**Figure 1:**
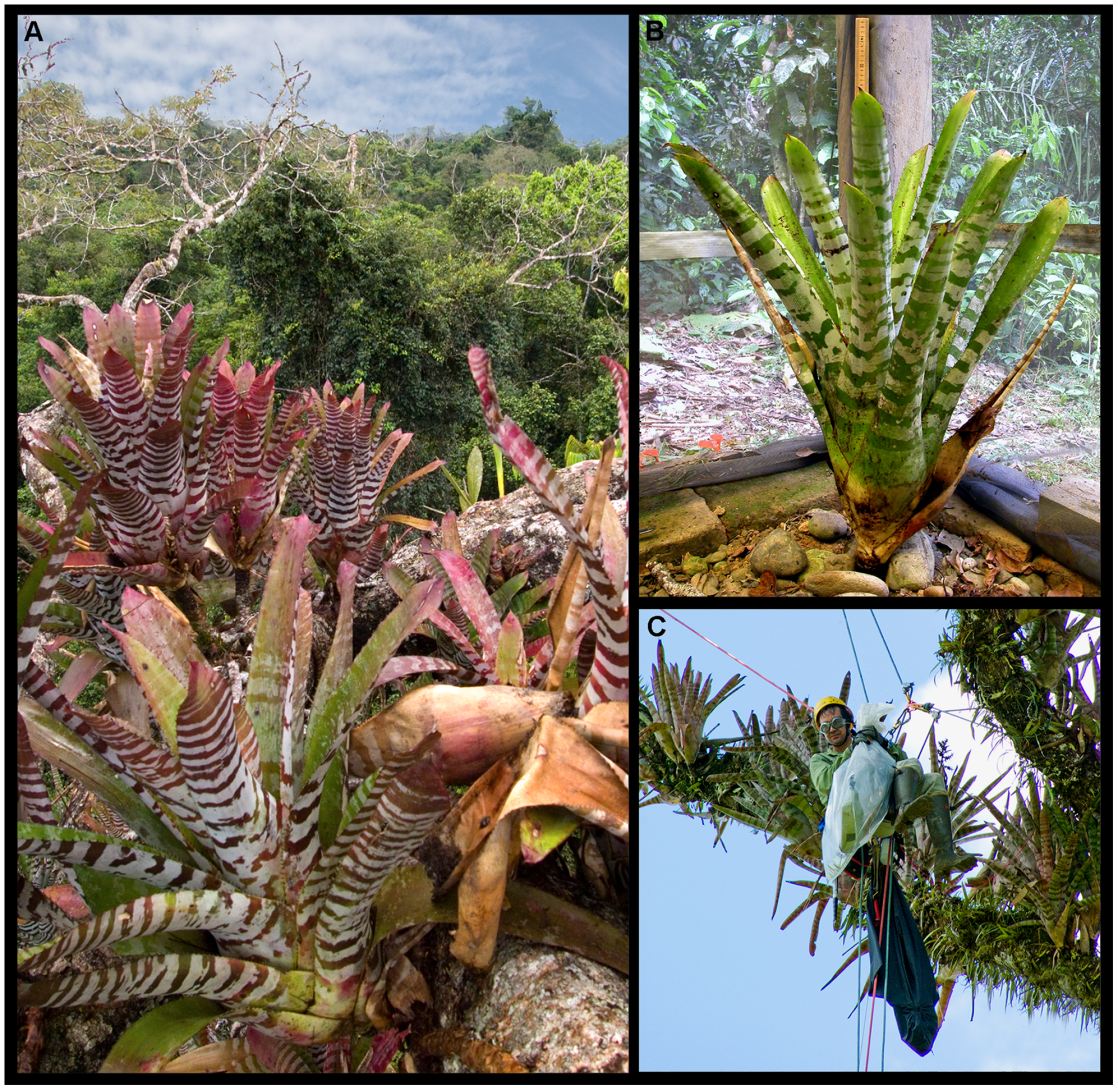
*Aechmea zebrina* tank bromeliads. (**A**) *Aechmea zebrina* (*in situ*) a large epiphytic tank bromeliad in the rainforest canopy of eastern Ecuador. (**B**) *A. zebrina* collected during bromeliad patch sampling. (**C**) Senior author using single-rope climbing technique to collect *A. zebrina* at 38 m in the canopy of a *Ceiba pentandra* tree.

We investigated occupancy and abundance of anurans inhabiting the epiphytic canopy tank bromeliad *Aechmea zebrina* (Smith) (Fig. 1), occurring in undisturbed and low-intensity disturbed lowland rainforest of the Yasuní Biosphere Reserve (Yasuní) in eastern Ecuador. In addition, habitat factors (e.g., host tree height and bromeliad tank water pH) were measured as potential predictors of anuran occupancy, abundance, and as correlates of undisturbed versus disturbed forest for *A. zebrina*. We tested for differences in *A. zebrina* anuran occupancy and abundance for measured factors between forest disturbance levels. We hypothesized that *A. zebrina* sampled along an oil access road edge with few forest clearings and a minimal footprint through primary forest (i.e. low-intensity disturbance) would reveal little to no impact on the anuran community, and oil roads with high-intensity disturbance driven by colonization would more likely show a negative effect on anurans. Due to bromeliad habitat loss in the high-intensity forest disturbance area during the study period we were unable to collect any data on anurans for this treatment level. Overall, we sought to determine the effects of oil roads and associated habitat modifications on the anuran inhabitants and habitat parameters of *A. zebrina* bromeliads in the upper canopy of an Amazonian lowland rainforest.

## Materials and Methods

### Ethics Statement

This study was carried out in strict accordance with the recommendations in the guidelines for use of live amphibians and reptiles in field research compiled by the American Society of Ichthyologists and Herpetologists (ASIH). Research was conducted in compliance to the rules overseen by the Texas State University Institutional Animal Care and Use Committee (Permit #: 0721-0530-7, 05-05C38ADFDB, and 06-01C694AF). Permission and permits issued by the Ministerio del Ambiente, Ecuador (Permit #: 006-IC-FA-PNY-RSO and 012-IC-FA-PNY-RSO).

### Study Area and Species

**Figure 2:**
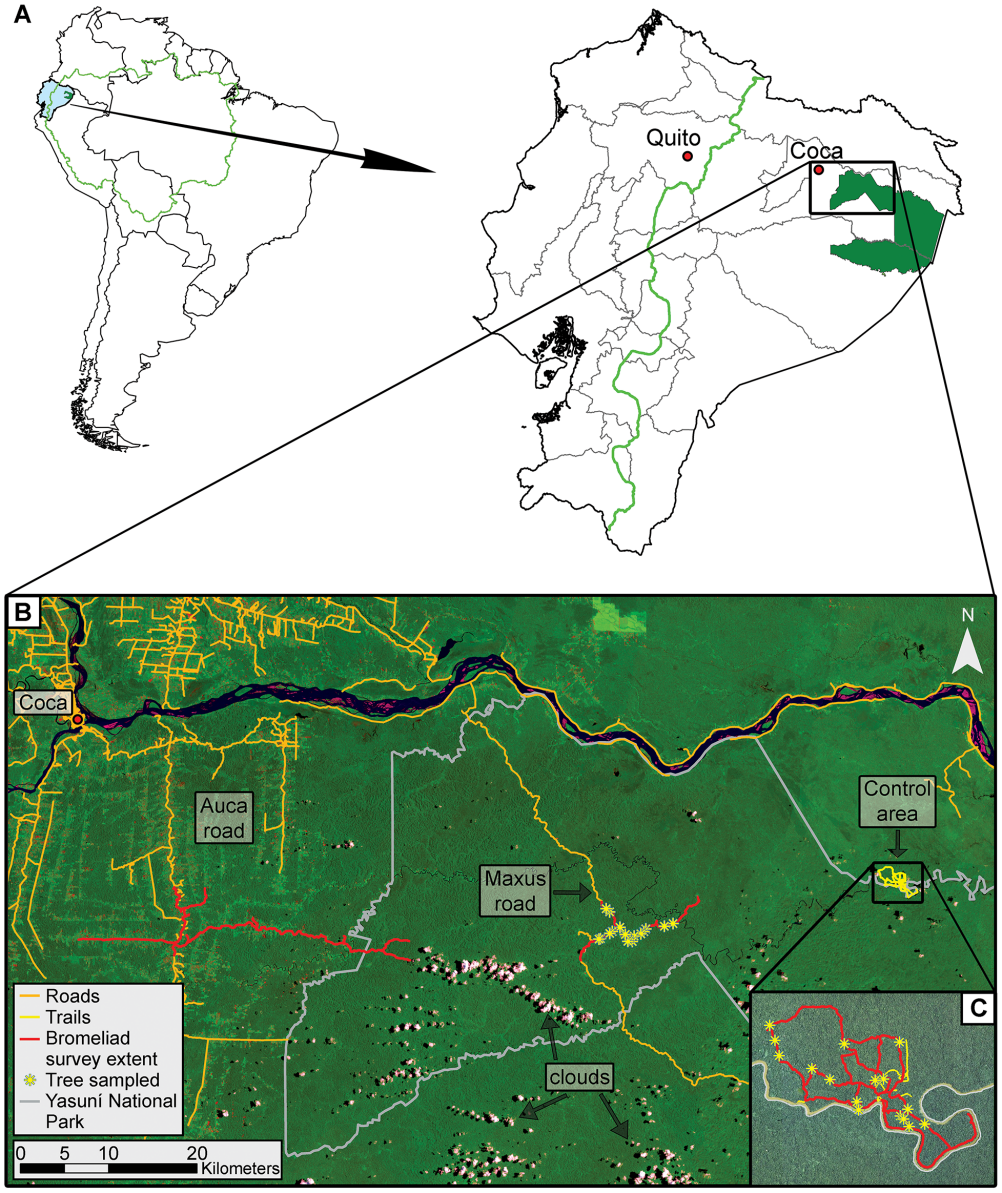
Map of the study area for *Aechmea zebrina* bromeliad host tree surveys and sampling. (**A**) Yasuní National Park (solid dark green) in the Amazon ecoregion (light green line) of eastern Ecuador. (**B**) 2004 Landsat ETM+ (bands 6,4, and 2) mosaic satellite image of study area surveyed for *A. zebrina* bromeliads and sampled trees, where the lightest and most brightly colored areas typically represent deforestation or secondary growth. Auca road = high-intensity forest disturbance, Maxus road = low-intensity forest disturbance, and Control area = undisturbed forest. (**C**) Detail of control area (undisturbed forest) for *A. zebrina* bromeliad surveys and sampling.

The study was conducted in the northwestern portion of Yasuní in Orellana Province, Ecuador. The biosphere (Yasuní) is composed of Yasuní National Park, Waorani Ethnic Reserve, and their respective buffer and transition zones [20] (Fig. 2). Yasuní covers ∼1.7 million ha of the Napo Moist Forests terrestrial ecoregion with an elevation range of 190–400 m above sea level [20], [36], [37]. The northwestern Yasuní region averages 2425–3145 mm of rainfall per year with no less than 100 mm per month [38], temperature averages 25°C (15°-38°C) [39], and humidity averages 88% [40]. Yasuní holds some of Ecuador's largest oil reserves, which is the country's primary export and accounts for the majority of government revenues [20], [36]. Oil operations are the primary driver of both direct and indirect causes of deforestation in Yasuní [20], [41]. Bass et al. [36] reported that Yasuní holds world record species diversity for several taxa, including the highest documented landscape scale herpetofauna diversity with 150 species of amphibians and 121 species of reptiles.

Trees sampled for *A. zebrina* bromeliads in low-intensity forest disturbance were located along the Maxus oil road system, and those sampled in undisturbed forest were located east of the road and separated by an average of 29 km (Fig. 2). The Maxus road was built in the early 1990s and is approximately 160 km in length. The Maxus is an unpaved gravel road averaging 6 m wide with managed low vegetation (<1 m) on each side at a width of 4 to 10 m. The maximum width of deforestation for the construction of the Maxus road was limited to approximately 25 m or less through the use of geotextiles as a road base instead of a traditional forest timber base [42], [43]. Most additional forest clearing is limited to the northern section of the road nearest the Napo River, where Kichwa colonists have begun practicing large-scale slash and burn agriculture [20]. The central and southern stretches of the road are occupied by a few small clusters of Waorani indigenous people who historically were semi-nomadic hunter-gatherers with small subsistence farms [20], [44]. Limited forest clearing has occurred along these roads even though the Waorani are more sedentary. The majority of cleared area is occupied by oil installations and infrastructure. However, the Waorani are beginning to adopt the Kichwa peoples agricultural practices for subsistence and market sales resulting in increased deforestation along the southern sections of road. Nonetheless these areas are still surrounded by large tracts of undisturbed forest. We performed additional survey work along an older network of oil roads, collectively known as the Auca road, which was to be used as a high-intensity forest disturbance treatment (Fig. 2). The decline of bromeliads during the course of this study prevented its inclusion in the analysis (details in Discussion).

*Aechmea zebrina* is a large epiphytic tank bromeliad, and relatively common in the lowland Amazon region of eastern Ecuador and southern Colombia (Fig. 1; Fig. 2). It can grow >1 m tall and wide, and hold nearly 4 L of water between its leaves (SFM, unpublished data). Typically, *A. zebrina* occurs in the upper canopy of overstory trees at vertical heights of 18–45 m, ranging in number of individuals from 1 to >150 on a single tree (SFM, unpublished data). *Aechmea zebrina* was chosen as the sampling unit in this study due to its relative abundance, large size, high number of individuals within a tree, previous confirmation of a diverse amphibian assemblage, and to control for any differences in inter-species community assemblages and microclimate [45].

### Study Design and Sampling Technique

We surveyed 24 km of trail in undisturbed forest to a distance of 50 m on each side, 24 km of road with low-intensity forest disturbance to a distance of 100 m on each side, and 50 km of road with high-intensity disturbed forest to a distance of 100 m on each side for all trees with communities of ≥15 *A. zebrina*. Sample trees were required to have a minimum of 15 *A. zebrina* so as not to decimate the community as a result of sampling. Coordinates for each tree were recorded with a global positioning system (Ashtech, Santa Clara, California) and distance from trail or road center was measured using a rangefinder (Nikon, Melville, New York). The distance from nearest road for trees in undisturbed forest was measured using Google Earth (Google, Mountain View, California). We graded trees for overall health and crown structure based on climbing safety and ease of access. We randomly selected 16 host trees per treatment for collection of 5 *A. zebrina*, totaling 80 bromeliads in each disturbance regime (Fig. 2). Tree surveys and bromeliad sampling were conducted during daylight hours between April and November of 2008. Yasuní’s rainfall and temperatures are typically described as aseasonal with January being the driest month but still receiving ≥100 mm of rain [36], [46], however we did not sample during this time period.

*Aechmea zebrina* were sampled following methods described by McCracken and Forstner [45]. We accessed the tree canopy using single-rope technique (SRT) (Fig. 1), and bromeliads were collected at estimated even vertical intervals between one another [47]. The number of *A. zebrina* inhabiting the tree was counted, and tree height and bromeliad elevation were recorded using a rangefinder. We removed bromeliads and sealed them in a 55-gallon (208 L) plastic bag before being lowered to the ground. We transported bromeliads back to camp and processed them in a screened tent to prevent escape of animals. Bromeliad water was poured through a 1-mm sieve to separate arthropods, leaf litter, and detritus. Water volume was measured with a graduated cylinder and pH with a 3-point calibrated pH probe (Oakton, Vernon Hills, Illinois). We measured the height of bromeliads to nearest centimeter and counted the number of mature leaves. We carefully dismantled each bromeliad leaf-by-leaf to collect all herpetofauna.

### Data Analyses

Before performing analyses, we conducted graphical data exploration to check for normality, homogeneity, and collinearity of explanatory variables [48], [49]. We used generalized linear mixed models (GLMMs) for occupancy, abundance, and habitat variable analyses of metamorphosed anurans inhabiting *A. zebrina* [50]. First, we performed analyses using the full dataset of all anuran species collected in *A. zebrina*. We then did analyses on two datasets with a reduced number of species, based on *a priori* knowledge about their use of canopy microhabitats. The reduced datasets consisted of 1) known obligate canopy-dwelling anurans (*Ranitomeya* spp. and *Scinax ruber* removed) and 2) only the two known obligate bromeliad-inhabiting anurans (*Pristimantis aureolineatus* and *P. waoranii*). For many species natural history data is uninformative as to whether these are obligate bromeliad-inhabitants, we therefore restricted the obligate bromeliad-inhabitant dataset to the two species (*Pristimantis aureolineatus* and *P. waoranii*) known as *Aechmea* spp. bromeliad specialists based on our previous bromeliad sampling (SFM, unpublished data). The use of GLMMs are ideal for ecological studies involving nonnormal data (i.e. binary or count data) with random effects, and allow models to be fit with appropriate error distributions for the response variable [48]. Incorporating random effects into the models accounts for the potential non-independence of subsampled data points in a nested design; and GLMMs fit with a Poisson distribution and individual-level random effect or negative binomial distribution without an individual-level random effect allow for overdispersion [48].

We used GLMMs to test for the effects of forest disturbance and recorded habitat variables on anuran occupancy of *A. zebrina* using a binomial error distribution, with a logit link function. Forest disturbance was coded as a binary variable and all other habitat variables were continuous. We used a similar model structure for anuran abundance and applied Poisson and negative binomial error distributions with a log link function. The R package “glmmADMB” provides the ability to fit Poisson and negative binomial error distributions with or without zero-inflation. Both of these distribution families and their variants have been shown to work best for count data [51], [52]. We took advantage of this capability to fit the full model and then conduct model reduction using the following error distributions: Poisson, zero-inflated Poisson, Poisson with individual-level random effect (log-normal Poisson), type 1 negative binomial (NB1, linear mean-variance relationship), zero-inflated NB1, type 2 negative binomial (NB2, quadratic mean-variance relationship), and zero-inflated NB2. We included the following habitat variables as fixed effects: forest disturbance, tree height, bromeliad elevation in tree, number of *A. zebrina* in tree, bromeliad height, number of mature bromeliad leaves, bromeliad water volume, and bromeliad water pH. Tank bromeliads are naturally replicated microcosms with physically discrete boundaries containing a distinct biotic community, allowing accurate measurement of both abiotic and biotic factors from an independent sampling unit [5], [53]–[56]. However, we treated each tree as if it were a random plot and consequently included tree as a random effect to address the potential non-independence (spatial correlation) of bromeliads sampled from the same tree (5 bromeliad samples nested within 16 trees for a total of 80 bromeliads sampled in each of two treatments).

We began the analysis with a full model containing all fixed and random effects, and their interactions with forest disturbance to test for differences in the effect of habitat variables between forest disturbance levels. For anuran abundance analyses we first identified the best-fit error distribution of the full model using the Akaike Information Criterion (AIC) before proceeding [57]. We carried out model reduction by first removing non-significant interaction terms and then main fixed effects using the AIC to determine best-fit models [57]. Only models within ΔAIC≤2 were considered similar to best-fit models for support of the data and reported herein [57]. Significance tests for fixed effects and their interactions were done using Wald *Z*-tests, which provide a more robust test than a likelihood ratio test when sample sizes are small [48]. We fit models with the laplace approximation using package “glmmADMB” (ver. 0.7.2.11) in R version 3.0.1 [51], [58].

## Results

We identified and mapped 56 trees in undisturbed forest (23 per 100 ha), 44 trees in low-intensity disturbed forest along oil roads (8 per 100 ha), and 0 trees in high-intensity disturbed forest along oil roads with ≥15 *A. zebrina*. On the Maxus road (low-intensity disturbance) there were a total of 6 oil operation facilities and 13 clearings by Waorani for home sites or crops along 24 km of surveyed road. The largest of these clearings extend approximately 100 m along the road with most being considerably smaller. These represent a small percentage of the 480 ha surveyed along the roads.

**Figure 3:**
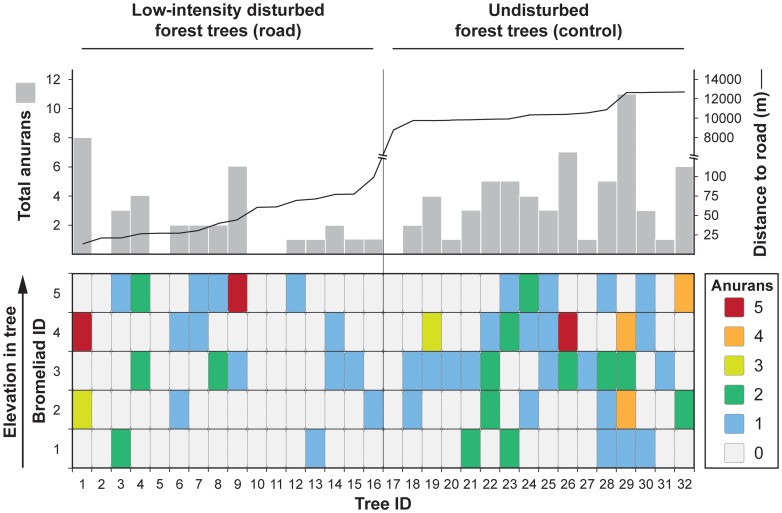
Anuran occupancy and abundance of sampled *Aechmea zebrina* bromeliads. Heat map of anuran occupancy and abundance sampled from *A. zebrina* in low-intensity disturbed forest and undisturbed forest. Trees are sorted by distance to road on the x-axis and bromeliads are sorted by elevation in tree on the y-axis. Summed anuran collections from all five bromeliads in each tree shown in upper bar graph with distance from road overlaid as line graph.

We collected 95 metamorphosed anurans representing 10 species from 160 bromeliads in 32 trees (Table 1). A total of 8 species were found in undisturbed and 5 in disturbed forest, with only 3 species shared. The rarity of most species did not allow for acceptable diversity index comparisons. Anurans were present in 36 bromeliads (45%) from 15 of 16 trees sampled in undisturbed forest, while 20 bromeliads (25%) from 12 of 16 trees were occupied by anurans in low-intensity disturbed forest (Fig. 3). The distance between a tree and nearest road was highly correlated with forest disturbance. Forest disturbance was our primary variable of interest, thus we elected to remove distance to road from GLMM analyses. Interaction terms included in all models to test for differences in recorded habitat variables between disturbance levels were not significant.

**Table 1:** Anuran species collected during bromeliad patch sampling, designated habitat, and abundance in disturbance levels.

Species name	Obligate canopy-dweller	Obligate bromeliad-inhabitant	Number in undisturbed forest	Number in disturbed forest
*Osteocephalus fuscifacies*	X		1	2
*Osteocephalus planiceps*	X		1	0
*Osteocephalus taurinus*	X		1	0
*Pristimantis acuminatus*	X		1	0
*Pristimantis aureolineatus*	X	X	23	13
*Pristimantis orphnolaimus*	X		2	0
*Pristimantis waoranii*	X	X	23	12
*Ranitomeya ventrimaculata*			0	1
*Ranitomeya variabilis*			9	0
*Scinax ruber*			0	6

### Anuran Occupancy Patterns

Only forest disturbance affected anuran occupancy in all models. In the full species dataset there were 44.4% fewer *A. zebrina* occupied by anurans in disturbed forest than undisturbed forest (β = −0.898, *Z* = −2.62, *p* = 0.009). The fixed factors for bromeliad elevation in tree, bromeliad water volume, and bromeliad water pH were retained in best-fit models within ΔAIC ≤2, but were not significant (Table 2). There was a 48.5% decrease in the number of anuran-occupied *A. zebrina* in disturbed forest for the reduced species dataset of obligate canopy-dwellers (β = −0.956, *Z* = −2.69, *p* = 0.007). The fixed factors of bromeliad water volume, number of *A. zebrina* in tree, bromeliad height, and bromeliad water pH were retained in best-fit models within ΔAIC ≤2, but were not significant. In the dataset for the two obligate bromeliad-inhabiting species there were 44.8% fewer *A. zebrina* occupied by anurans in disturbed forest than undisturbed forest (β = −0.828, *Z* = −2.21, *p* = 0.027). The number of *A. zebrina* in tree and bromeliad water pH were the only non-significant fixed factors retained in best-fit models within ΔAIC ≤2 for the obligate bromeliad-inhabitants.

**Table 2:** Best-supported models (ΔAIC≤2) for anuran occupancy of *A. zebrina* bromeliads for datasets containing all species, obligate canopy-dwellers, and obligate bromeliad-inhabitants.

Dataset	Model^d^	Fixedeffects	β^e^	SE	Z	P	ΔAIC
Full^a^	f1bin	Intercept	−0.201	0.225	−0.89	0.372	–
		Forestdisturbance	−0.898	0.342	−2.62	0.009*	
	f2bin	Intercept	−0.565	0.424	−1.33	0.183	1.0
		Forestdisturbance	−0.892	0.343	−2.60	0.009*	
		Water volume	<0.001	<0.001	1.02	0.310	
	f3bin	Intercept	0.491	1.028	0.48	0.630	1.7
		Forestdisturbance	−0.814	0.351	−2.32	0.020*	
		*A. zebrina* elevation	−0.036	0.032	−1.12	0.260	
		Water volume	<0.001	<0.001	1.20	0.230	
Canopy^b^	c2bin	Intercept	0.060	0.364	0.16	0.869	–
		Forestdisturbance	−0.937	0.358	−2.62	0.009*	
		Number of*A. zebrina*	−0.007	0.005	−1.43	0.152	
	c1bin	Intercept	−0.354	0.227	−1.56	0.120	0.1
		Forestdisturbance	−0.956	0.355	−2.69	0.007*	
	c3bin	Intercept	−0.365	0.484	−0.75	0.450	0.2
		Forestdisturbance	−0.917	0.360	−2.55	0.011*	
		Water volume	<0.001	<0.001	1.34	0.181	
		Number of*A. zebrina*	−0.008	0.005	−1.67	0.096	
	c4bin	Intercept	0.622	1.598	0.39	0.697	1.8
		Forestdisturbance	−0.921	0.360	−2.56	0.011*	
		Water volume	<0.001	<0.001	1.34	0.179	
		Number of*A. zebrina*	−0.008	0.005	−1.73	0.084	
		Water pH	−0.218	0.337	−0.65	0.518	
Bromeliad^c^	b1bin	Intercept	−0.570	0.242	−2.35	0.019	–
		Forestdisturbance	−0.828	0.374	−2.21	0.027*	
	b2bin	Intercept	−0.261	0.374	−0.70	0.049	0.9
		Forestdisturbance	−0.807	0.371	−2.17	0.030*	
		Number of*A. zebrina*	−0.005	0.005	−1.03	0.300	
	b3bin	Intercept	1.237	1.622	0.76	0.446	2.0
		Forestdisturbance	−0.820	0.375	−2.19	0.029*	
		Number of*A. zebrina*	−0.005	0.005	−1.12	0.261	
		Water pH	−0.331	0.351	−0.94	0.345	

^a^ Dataset analyzed with all anurans collected from *A. zebrina* bromeliads.

^b^ Dataset analyzed using only obligate canopy-dwelling anurans.

^c^ Dataset analyzed using only obligate bromeliad-inhabiting anurans.

^d^ All models analyzed using binomial error distribution.

^e^ Coefficient estimate.

Statistically significant effects (p<0.05).

### Anuran Abundance Patterns

Similarly, only forest disturbance affected anuran abundance. The best-fit model for the full species dataset, as determined by AIC, was based on the log-normal Poisson distribution. However, we report the model based on the type 1 negative binomial distribution because it was within ΔAIC ≤2, was more parsimonious for number of factors used, and because both models produced similar results (Table 3). Anuran abundance in the full species dataset was 44.3% less in disturbed forest compared to undisturbed forest (β = −0.792, *Z* = −2.61, *p* = 0.009). No other factors were retained in either best-fit model for the full dataset. The best-fit model for the obligate canopy-dwellers dataset was based on the log-normal Poisson distribution with forest disturbance level as the only retained fixed factor, but 3 other models were within ΔAIC ≤2 and reported mixed significance effects for forest disturbance level (Table 3). There were 48% fewer obligate canopy-dwelling anurans in disturbed forest than in undisturbed forest (β = −0.723, *Z* = −2.11, *p* = 0.035). In the dataset for obligate bromeliad-inhabiting anurans the best-fit model was based on the log-normal Poisson distribution with forest disturbance level as the only retained fixed factor, but 2 other models were within ΔAIC ≤2 and reported similar results (Table 3). There were 44.8% fewer obligate bromeliad-inhabiting anurans in disturbed forest than undisturbed forest (β = −0.672, *Z* = −1.77, *p* = 0.076).

**Table 3:** Best-supported models (ΔAIC≤2) of anuran abundance in *A. zebrina* bromeliads for all species, obligate canopy-dwellers, and obligate bromeliad-inhabitants.

Dataset	Model	Error distribution^d^	Fixed effects	β^e^	SE	Z	P	ΔAIC
Full^a^	f1lnp	LN Poisson	Intercept	−0.897	0.253	−3.54	<0.001	–
			Forest disturbance	−0.801	0.329	−2.44	0.015*	
	f1nb1	T1 Neg. Bin.	Intercept	−0.373	0.204	−1.83	0.068	2.0
			Forest disturbance	−0.792	0.303	−2.61	0.009*	
Canopy^b^	c1lnp	LN Poisson	Intercept	−0.943	0.263	−3.58	0.000	–
			Forest disturbance	−0.723	0.342	−2.11	0.035*	
	c2lnp	LN Poisson	Intercept	−0.555	0.365	−1.52	0.013	0.0
			Forest disturbance	−0.699	0.335	−2.09	0.037*	
			Number of *A. zebrina*	−0.006	0.004	−1.40	0.163	
	c3lnp	LN Poisson	Intercept	−1.440	0.760	−1.90	0.058	0.2
			Forest disturbance	−0.600	0.332	−1.81	0.071	
			Number of *A. zebrina*	−0.007	0.004	−1.61	0.108	
			*A. zebrina* leaf number	0.032	0.024	1.35	0.175	
	c4lnp	LN Poisson	Intercept	−2.033	1.061	−1.92	0.055	1.6
			Forest disturbance	−0.656	0.341	−1.93	0.054	
			Number of *A. zebrina*	−0.008	0.005	−1.77	0.077	
			*A. zebrina* leaf number	0.029	0.024	1.23	0.219	
			*A. zebrina* elevation	0.024	0.303	0.80	0.422	
Bromeliad^c^	b1lnp	LN Poisson	Intercept	−1.178	0.300	−3.92	<0.001	–
			Forest disturbance	−0.672	0.379	−1.77	0.076	
	b2lnp	LN Poisson	Intercept	−2.009	0.823	−2.44	0.015	0.8
			Forest disturbance	−0.592	0.377	−1.57	0.117	
			*A. zebrina* leaf number	0.029	0.026	1.11	0.265	
	b3lnp	LN Poisson	Intercept	−1.767	0.840	−2.10	0.035	1.1
			Forest disturbance	−0.548	0.369	−1.48	0.138	
			Number of *A. zebrina*	−0.006	0.005	−1.29	0.196	
			*A. zebrina* leaf number	0.034	0.026	1.31	0.191	

^a^ Dataset analyzed with all anurans collected from *A. zebrina* bromeliads.

^b^ Dataset analyzed using only obligate canopy-dwelling anurans.

^c^ Dataset analyzed using only obligate bromeliad-inhabiting anurans.

^d^ Best-fit error distribution as determined by AIC; LN Poisson = Log-normal Poisson, T1 Neg. Bin. = Type 1 negative binomial.

^e^ Coefficient estimate.

Statistically significant effects (p<0.05).

## Discussion

### Disturbance Effects on Anuran Assemblages

Our results show that forest disturbance associated with oil access roads and infrastructure has a negative effect on anurans utilizing the microhabitat of *A. zebrina* bromeliads in the upper canopy of eastern Ecuador’s lowland rainforest. *Aechmea zebrina* bromeliads in low-intensity disturbed forest along the Maxus oil roads had significantly lower occupancy by anurans than in undisturbed forest, with nearly twice as many *A. zebrina* occupied by one or more anurans in undisturbed forest. In low-intensity disturbed forest we found a significantly lower abundance of anurans in *A. zebrina* for both the entire community and canopy-dweller datasets. While differences in abundance for the obligate bromeliad-inhabitants were not significant, they did follow a similar trend with about half as many anurans being observed in low-intensity disturbed forest. This is consistent with the full dataset where there was a fractionally smaller reduction in percent abundance of anurans (44.3%) in low-intensity disturbed forest compared to the obligate bromeliad-inhabitants dataset (44.8%). The magnitude of these results was unexpected due to the relative intactness of the forest along the roads.

The limited studies of terrestrial amphibians in Neotropical lowland rainforest associated with forest clearing, fragmentation, or edge effect have generally reported negative effects on amphibian community diversity and abundance [59]–[61]. However, these anthropogenic disturbances sometimes have positive or neutral effects when the focus is on particular species groups (e.g., Hylidae) [60], [62]. Typically, anthropogenic disturbance effects are correlated with habitat differences among disturbance levels or types (e.g., soil moisture, distance from clearing or edge, and leaf litter depth) [59], [60], [63]. In contrast, Ernst and Rödel [64] found tropical tree frog assemblages were affected by disturbance regime and geographic distance but habitat factors were not significant predictors of species incidence, even when habitat factors differed between disturbance regimes [64], [65]. We similarly found no relationship between anuran occupancy or abundance and habitat factors, but in our case the habitat variables did not differ between undisturbed and low-intensity disturbed forest. Several studies have yielded contrasting results for different anuran assemblages (i.e. leaf litter, canopy, and stream communities) in relation to environmental (including habitat), spatial, and spatially structured environmental effects [33], [64]–[68]. Different anuran assemblages are subject to different structuring forces making it difficult to identify correlated factors, particularly in poorly studied assemblages [68].

### Disturbance Effects on *A. zebrina* Bromeliads

The initial sampling design for this study included a high-intensity disturbance area along an older network of oil roads known as the Auca road. A preliminary survey, conducted in August 2006, yielded trees with *A. zebrina* suitable for sampling along the central portion of the Auca road (Fig. 2). The Auca road is heavily deforested and fragmented due to uncontrolled colonization; the majority of road frontage has been cleared for crops and pasture with other colonizers moving in behind these farms, resulting in a quasi-parallel pattern of deforestation and fragmentation [69]. Upon return for sampling in 2008, trees with *A. zebrina* no longer existed along either side of the roads, including a section that extends into Yasuní within 20 km of trees sampled along the Maxus oil road. Herbarium records and other road surveys confirm the presence of *A. zebrina* from throughout the surrounding region (SFM, unpublished data). A light aircraft flight was taken by SFM on November 15, 2008 which crisscrossed the Auca road region to search for *A. zebrina* in emergent trees within areas of remaining intact forest that were not accessible from road surveys. During this flight 15 trees with *A. zebrina* communities were identified, the majority being greater than a kilometer from the nearest road and the closest approximately 450 m from a road that showed no signs of clearing in the immediate area. Although somewhat anecdotal, evidence from road and aerial surveys indicate that *A. zebrina* are intolerant of deforestation but to a lesser extent forest fragmentation.

Along the Maxus oil road where we conducted our sampling, most stretches of the road have primary forest up to the right-of-way edge on either side. However, it does appear that the cumulative effects of deforestation and fragmentation along roads may be having a negative impact on the occurrence of trees with *A. zebrina* communities based on our road census results. The Maxus oil road is the most strictly controlled in eastern Ecuador, minimizing non-indigenous settlers for more than 15 years [20]. The fact that no recorded habitat variables differed between disturbance levels is another indicator of the quality of forest bordering the Maxus oil road.

### Road Impacts on Canopy Climate and Biota

In the absence of habitat correlations with anuran occupancy or abundance, and no differences in these variables between forest disturbance levels it is difficult to explain the observed negative effect on anurans inhabiting high canopy *A. zebrina* along an oil road. Linear clearings, such as roads, result in edge-related changes to forest structure, microclimate, and forest dynamics that often penetrate up to 200 m into the adjacent forest with some effects detectable up to 500 m from the edge [21], [22]. For example, Pohlman et al. [70] found greater diurnal fluctuations in light, temperature, and humidity in forests within 50–100 m of edges, with such areas being typically drier and hotter than forest interiors further from those edges. A positive relationship exists between the width of linear clearings and the intensity of edge effects, particularly when clearing width is greater than 20 m [71]. Edge-related changes in climatic factors and increased wind disturbance elevate desiccation stress and are associated with greater tree mortality than found for interior forests [71], [72]. Most observations of forest disturbance effects on habitat and climatic factors have been collected near ground level [22], [59]–[61], [65]–[67], [70]. Disturbance effects are likely elevated in the canopy where the forest interfaces with the atmosphere and the fluctuation of climatic factors are more extreme [26], as compared to the lower forest strata which benefits from climate moderating effects of the canopy [73].

Epiphytes, including bromeliads, contribute significantly to maintaining microclimatic conditions in tropical forest canopies by reducing wind turbulence and temperature which, in turn, reduces evapotranspiration and helps maintain elevated relative humidity [74], [75]. Epiphytes are considered hypersensitive to changes in climatic conditions [66], [75], [76]. This hypersensitivity makes them particularly susceptible to forest microclimate changes resulting from anthropogenic disturbance, thus epiphytes and their inhabitants are suitable bioindicators of biodiversity and forest integrity [76]–[79]. While it is generally accepted that bromeliads contribute to canopy microclimate moderation, basic knowledge of the internal microclimate factors supporting the diverse and abundant faunal communities within the bromeliad microhabitat is scarce [8], [77], [80]. Canopy perturbations, whether anthropogenic or natural, can alter microclimate conditions in forest canopies causing disruptions to ecosystem functions and biodiversity, with local to global effects on climatic variables such as increased temperatures and reduced precipitation [2], [81].

Disruption of canopy microclimate is a potential driver for reduced presence and abundance of anurans in disturbed forest bromeliads. Additionally, it may be the explanation for a reduced number of trees with large (≥15) *A. zebrina* communities. The combination of a reduction in habitat availability and connectivity with poor microclimate conditions within the remaining habitat may have shifted canopy anurans to more suitable habitat away from the roads. However, we did observe *A. zebrina* closest to the road with the highest anuran abundances were also in close proximity to oil operation facilities. We collected *Scinax ruber*, a disturbance specialist and invasive canopy anuran, in *A. zebrina* sampled from two trees adjacent to oil operation facilities which accounted for 18% of total anuran observations in low-intensity disturbed forest (Text S1). A possible explanation for these observations is the reduced habitat availability, which may cause clustering of species in remaining habitat. In addition, the proximity to artificially lighted facilities creating an increase in prey items and reduced primate predators [82], [83], may lead to an increased concentration of invasive anurans in the remaining bromeliads.

Another explanation may be pollution from road dust or waste gas flaring at oil facilities entering the bromeliad tank water and directly poisoning anurans or disrupting the food web. Road dust from large trucks and heavy equipment traveling the Maxus oil road has been observed penetrating the canopy at elevations of approximately 35 m and a distance of 26 m from the road edge (SFM, personal observation). Road noise and vibrations have also been found to impact some organisms including amphibians [42], [84]. The mean distance of 29 km separating undisturbed and disturbed forest sampling locations helped ensure that oil operations, hunting, and forest product harvesting by Waorani and Kichwa peoples conducted in the vicinity of the oil road would not affect bromeliads sampled in undisturbed forest. Hunting and forest product harvest, including *A. zebrina* harvest by Kichwa for medicinal and ornamental purposes [76], may have a negative impact on bromeliad-inhabiting anurans due to a reduction in habitat and possibly canopy predators. Canopy mammals and birds forage in bromeliads and primate predation of an anuran in *A. zebrina* has been observed in undisturbed forest (SFM, personal observation). Waorani and Kichwa hunting in forest bordering the oil road is causing a decline in mammal populations, and this reduction in mammalian predators (primarily primates) may have a positive impact on anurans inhabiting the canopy [44], [83], [85]. Anthropogenic disturbance, particularly roads, has negatively impacted a number of tropical biotic groups including trees, epiphytes, terrestrial and arboreal mammals, birds, amphibians, reptiles, fishes, and invertebrates [21], [22], [70], [71], [84]–[87]. A variety of negative effects caused by roads and linear clearings have been attributed to these impacts on tropical biota [21], but teasing apart how these effects impact particular groups and species assemblages remains a challenge to ecologists.

## Conclusions

Our study provides an extensive look into the anuran fauna of a large upper canopy tank bromeliad and the unexpected negative effects of low-intensity forest disturbance due to a road edge with limited deforestation. While our study focuses on the anuran inhabitants and a selection of habitat parameters for one species of canopy bromeliad, there are many more canopy bromeliads and organisms dependent on this aquatic resource in the harsh canopy environment. We suggest several avenues of research for future studies to better understand the impacts on canopy diversity resulting from what is considered to be limited forest disturbance by natural resource extraction operations. First, more data collection and analyses of bromeliad microhabitat and inhabitants for presence, abundance, and diversity associated with deforestation and fragmentation area. Second, more detailed monitoring of microclimate factors within and adjacent to upper canopy bromeliads at stratified levels of forest disturbance to determine correlates of differences. Third, bromeliad tank water quality testing for pollutants deposited with road dust, generated as part of the petroleum refining processing, and from agricultural burning. Further canopy research would provide us with a better understanding of how tropical forest canopy ecosystems may be disrupted by direct and indirect human activities that result in cascading effects right down to the forest floor.

The detrimental effects of petroleum operations on tropical forests have been well documented in eastern Ecuador and around the world [19], [41]. While further research is needed, our study reveals that even a minimal environmental footprint by petroleum extraction operations, primarily roads, can have significant impacts on a unique anuran community in perhaps the most biologically diverse place on the planet [36]. Based on these results, we support the recommendations of Bass et al. [36] to permit no new terrestrial access routes into Yasuní or its buffer zone and establish a moratorium on future exploration and extraction operations. A strategy for minimizing negative effects of petroleum development on native flora and fauna would 1) employ industry best practices guidelines [88], 2) treat tropical forest habitat as an offshore system where land-based access is not used, and 3) protect current access roads from further colonization and subsequent deforestation.

## Supporting Information

Text S1 **An anuran canopy bromeliad invader,** ***Scinax ruber.***
(DOCX)
