# Supplementary material for: Oil Road Effects on the Anuran Community of a High Canopy Tank Bromeliad (Aechmea zebrina) in the Upper Amazon Basin, Ecuador
Source: PLoS One. 2014 Jan 8;9(1):e85470. doi: 10.1371/journal.pone.0085470 (PMC3885719; doi:10.1371/journal.pone.0085470)
Supplement: Text S1 — An anuran canopy bromeliad invader, Scinax ruber. (DOCX) [file pone.0085470.s001.docx]

Supporting Information: Text S1

An anuran canopy bromeliad invader, *Scinax ruber*

While we did not analyze abundance-based indices of species diversity, it was the combination of several species represented as singletons or doubletons and nearly twice the number of obligate bromeliad-inhabitants in undisturbed forest that contributed to differences in forest disturbance treatments. *Scinax ruber* was one species of particular interest found only in disturbed forest trees in close proximity to a large oil production facility, the largest cleared and developed site in our study area. *S. ruber* is arboreal and considered a “pest” species because of its invasiveness and propensity to displace native species [1]. Its primary habitat is disturbed or cleared areas and is not considered a bromeliad or upper canopy inhabitant [2], although Pauly *et al.* [3] reported observations of *S. ruber* on a canopy tower in Panama at heights up to 25 m. The canopy tower is in close proximity to the Panama Canal and the town of Gamboa, an area of historic and current anthropogenic disturbances and most likely reason for its presence. In this study and previous work in undisturbed forest areas of Yasuní (130 bromeliads from 3 species and 140 quadrat surveys) we have not collected *S. ruber*, yet it has been observed around the few buildings in camp areas (SFM, personal observation). Therefore, we do not consider *S. ruber* an upper canopy bromeliad-inhabitant; rather, it is an invasive species to the upper canopy near areas of heavy human disturbance where its populations have exploded. Linear clearings (i.e. roads and pipelines) are particularly efficient at facilitating faunal invasions [4], like that of *S. ruber*, as a result of their significant increase in disturbed edge size and deep penetration into forest habitat. Faunal and floral invasions by non-native species following roads have already penetrated remote areas of tropical forests causing disruptions to species assemblages, displacement or localized extirpation of native species, alongside the introduction of novel and potentially lethal pathogens [4], [5].

Literature Cited

1. Solís F, Ibáñez R, Jaramillo C, Fuenmayor Q, Azevedo-Ramos C, et al. (2011) IUCN 2011. Gland: IUCN Red List of Threatened Species.

2. Duellman WE (1978) The biology of an equatorial herpetofauna in Amazonian Ecuador. Misc. Pub. Mus. Nat. Hist. Univ. Kansas 65: 1–352.

3. Pauly GB, Bernal X, Taylor RC (2005) *Scinax ruber* - arboreality and parachuting. Herpetol. Rev. 36: 308–309.

4. Goosem M (1997) Internal fragmentation: the effects of roads, highways, and powerline clearings on movements and mortality of rainforest vertebrates. In: Laurance WF, Bierregaard RO, editors. Tropical forest remnants, ecology, management, and conservation of fragmented communities. Chicago: The University of Chicago Press. pp. 241–255.

5. Laurance WF, Goosem M, Laurance SGW (2009) Impacts of roads and linear clearings on tropical forests. Trends Ecol. Evol. 24: 659–669.
